# Human and Murine Kidneys Show Gender- and Species-Specific Gene Expression Differences in Response to Injury

**DOI:** 10.1371/journal.pone.0004802

**Published:** 2009-03-11

**Authors:** Han Si, Ramandeep S. Banga, Pinelopi Kapitsinou, Manjunath Ramaiah, Janis Lawrence, Ganesh Kambhampati, Antje Gruenwald, Erwin Bottinger, Daniel Glicklich, Vivian Tellis, Stuart Greenstein, David B. Thomas, James Pullman, Melissa Fazzari, Katalin Susztak

**Affiliations:** 1 Department of Medicine/Nephrology, Albert Einstein College of Medicine, Bronx, New York, United States of America; 2 Department of Surgery, Montefiore Medical Center, Bronx, New York, United States of America; 3 Department of Pathology, Montefiore Medical Center, Bronx, New York, United States of America; 4 Department of Medicine/Nephrology, Mount Sinai School of Medicine, New York, United States of America; 5 Department of Epidemiology and Population Health, Albert Einstein College of Medicine, Bronx, New York, United States of America; University of Texas Arlington, United States of America

## Abstract

The incidence of End Stage Renal Disease (ESRD) is approximately 50% higher in men than women. In order to understand the molecular basis of this gender disparity, we examined sex specific gene expression patterns in control and diseased, human and murine kidney samples. Using the Affymetrix platform we performed comprehensive gene expression analysis on 42 microdissected human kidney samples (glomeruli and tubules). We identified 67 genes with gender biased expression in healthy human kidneys and 24 transcripts in diseased male and female human kidneys. Similar analysis performed in mice using male and female control and doxorubicin induced nephrotic syndrome kidneys identified significantly larger number of differentially expressed transcripts. The majority of genes showing gender biased expression either in diseased human and murine kidneys were different from those differentially expressed in healthy kidneys. Only 9 sexually dimorphic transcripts were common to healthy human and murine kidneys and five showed differential regulation in both human and murine diseased kidneys. In humans, sex biased genes showed statistical enrichment only to sex chromosomes while in mice they were enriched to sex chromosomes and various autosomes. Thus we present a comprehensive analysis of gender biased genes in the kidney. We show that sexually dimorphic genes in the kidney show species specific regulation. Our results also indicate that male and female kidneys respond differently to injury. These studies could provide the basis for the development of new treatment strategies for men and women with kidney disease.

## Introduction

Chronic kidney disease (CKD) affects about 20 million adults and it is a cause of significant morbidity and mortality in the United States [1], [2], [3]. Male gender affects the prevalence and progression of renal disease and it is associated with a 50% higher incidence of ESRD. A large meta-analysis (including 11,345 patients from 68 studies) performed recently indicates that patients with autosomal dominant polycystic kidney disease (PKD), IgA nephropathy, membranous nephropathy, or chronic renal disease of unspecified etiology progress more quickly to ESRD [4]. The difficult and clinically important question would be to determine whether these gender differences are due to “environmental” co-variants, sex hormone differences, inherent functional and anatomical differences in male and female kidneys or due to their differential injury response.

Similarly, in most animal models of chronic renal disease (hypertension, renal ablation models and PKD), males show accelerated progression of renal injury compared to females [5], [6]. Animal studies suggest that sex hormones per se, rather than genetically determined structural differences, cause the greater susceptibility of male kidneys to progressive renal injury. Hormonal manipulation studies suggest that female sex hormones such as estradiol may slow the progression of renal disease, whereas male hormones such as testosterone may promote disease progression [7], [8]. The biological effects of sex steroid hormones are classically mediated by nuclear receptors. They either act as ligand-activated transcription factors that bind to specific response elements located on the promoters of target genes, or by tethering to transcription factor complexes that contact DNA at alternative sites [9], [10]. Sex steroid hormones can also exert rapid non-genomic effects including the activation of the MAP kinase pathways [11], [12].

In addition, to the effects mediated by sex-steroid hormones, there is a possibility that the expression of sex chromosomal genes might also contribute to sexual dimorphisms [13]. During development, the presence of Y chromosome is critical for male specification. To restore the balanced expression of X-linked genes between sexes, gene dosage compensation mechanisms have evolved. In females, large parts of one X-allele are silenced by X-inactivation reducing gene dosage to male level, but some genes escape X-inactivation [14], [15], [16], [17]. It is also possible that, higher expression level of X-chromosome genes in females might be compensated by the expression of functionally equivalent Y-chromosome genes in males. The human Y chromosome encodes >100 genes, while only 23 protein coding genes have been annotated on the murine Y-chromosome so far, indicating that sexual dimorphisms of sex-chromosome-linked genes might be species-specific. Y-chromosome genes have mainly been studied in the context of male fertility and for the most part their role and regulation is unknown [18].

Rinn *et al.* [19] performed one of the first gene expression studies using Affymetrix arrays to determine whole genome scale expression differences between male and female Swiss-Webster mice. They found that the degree of sexual dimorphism in different organs show large variations. Very few genes showed differential expression in the hypothalamus, while the kidney had the most sex-biased transcripts. The study by Rinn et al. identified 27 sexually dimorphic genes in murine kidneys with at least >3 fold (p<0.001) change [20]. The dominant classes of differentially expressed genes detected in the study were monooxygenases, involved in electron transport (including the biosynthesis and metabolism of eiconasoids), transporters in the solute carrier family and glucuronosyltransferases. The authors concluded that genes involved in drug metabolism and renal function represent the major molecular differences between mammalian sexes. A recently performed comprehensive microarray analysis (using the Agilent platform) of 334 mice (an F2 intercross between C57Bl/6J and C3H/heJ strains) proposed that the extent of sexual dimorphism in gene expression is greater than previously recognized [21]. Sex-biased expression was detected for 72% (liver), 68% (adipose), 55% (muscle) and 13.6% (brain) of all active genes (kidneys were not examined). The expression of sex biased genes was highly tissue-specific and was enriched not only on the sex chromosomes, but also on chromosome 7, 13 and 19.

Given the high degree of baseline sexual dimorphism in the kidney and the significant gender differences in renal disease development, a systematic analysis of gender differences is of great importance. Such analysis could provide insight into the mechanism responsible for sex differences in renal disease progression, drug metabolism and response. In addition, most, if not all, large-scale gene expression studies to identify gender specific gene expression pattern have been performed in rodents under baseline condition. Sexually dimorphic gene expression in renal diseases has not been analyzed. We also increasingly recognize that gene expression studies performed in rodents cannot be fully translated into humans, and that many genes show discordant regulation in humans compared to rodents. To address these issues we performed whole genome scale expression studies in healthy and diseased human kidney samples and mouse models of renal disease.

## Results

### Gene Expression Differences in Healthy and Diseased Mouse Kidneys

**Figure 1:**
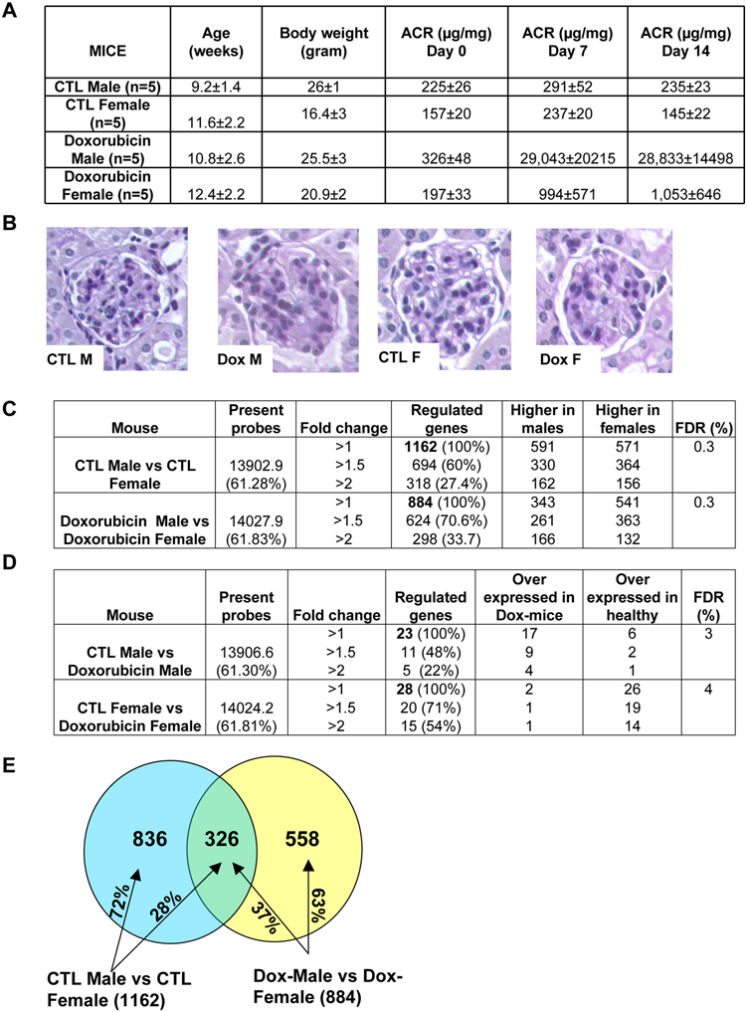
Phenotype and gene expression differences in doxorubicin injected male and female Balb/c mice. (A) Phenotypic description of the animals used in the study (B) PAS staining of murine kidney sections. CTL-control, dox-14 days following doxorubicin injection, M-male, F-female (C) The number of genes that are differentially expressed in kidneys between male (M) mice and female (F) mice in control (CTL) and diseased (dox) condition induced by doxorubicin. The value in parenthesis shows the percentage of active genes or the percent of all regulated genes. (D) Gene expression regulation between control and diseased male mice, control and diseased female mice (SAM analysis, FDR; false discovery rate). (E) Overlap of sexually dimorphic genes in healthy and diseased condition.

We used AffymetrixM430A2.0 expression arrays to determine global gene expression differences in kidneys of 10-week old healthy Balb/c male and female mice (n = 5/group, Figure 1A). Phenotypic description of the animals is shown on Figure 1. As expected the body weight of male and female mice was different and there was a minor but statistically significant difference in baseline albuminuria (Figure 1A). We used the Significance Analysis of Microarray data (SAM) [22] with a stringent false discovery rate (FDR) of 0.3% and we identified 1,162 differentially expressed transcripts between male and female kidneys. The differentially expressed genes are listed in Supplemental Table S1. The difference in gene expression level was relatively small and half of the transcripts (468) showed less than 50% change in their expression level (Figure 1C). (There was an equal distribution in the differentially expressed genes that showed higher expression level in males or females (Figure 1C).)

In order to identify gene expression differences in response to injury, animals were injected with doxorubicin, a substance that is known to induce albuminuria and glomerulosclerosis in Balb/c mice as it is directly toxic to podocytes [23]. Male mice developed albuminuria as early as 7 days following the administration of doxorubicin and albumin creatinine ratio (ACR) was as high as 28,833±14,498 µg/mg on day 14. The degree of albuminuria was lower in female mice (ACR of 1,053±646 µg/mg)(Figure 1A). In conjunction, the degree of glomerulosclerosis was also greater in male mice compared to female mice (Figure 1B). To understand the molecular mechanism of this differential injury response we analyzed gene expression changes in diseased male and female kidneys. The SAM analysis (FDR 0.3%) identified 884 differentially expressed transcripts when we compared gene expression profiles in diseased male and female kidneys (Figure 1C and Table S2). We determined that 326 transcripts showed sexual dimorphism in both healthy and diseased kidneys (Figure 1E). We also compared gene expression differences in control vs. doxorubicin injected male mice and identified 23 differentially expressed transcripts (Figure 1D, Table S3). Similar analysis comparing control vs. doxorubicin injected female animals identified 28 differentially expressed genes (Figure 1D, Table S3). Strikingly, no transcripts were found to be commonly differentially regulated during the disease progression in males and females. The absence of overlap indicates a differential injury pattern in male and female mice (this analysis was first filtered for differentially expressed genes within the same gender). The expression of the top differentially regulated genes was confirmed by QRT-PCR and relative gene expression levels showed excellent correlation (r^2^∼0.9) to the microarray results (data not shown).

### Gene Expression Differences in Healthy and Diseased Human Kidneys Show Sexual Dimorphism

Next we analyzed sex biased gene expression differences in control and diseased human kidneys. We collected 42 kidney samples from healthy living transplant donors, nephrectomies and from diagnostic kidney biopsies. Preliminary studies did not show significant gene expression differences in control kidneys based on the collection method (i.e. living kidney biopsy vs. unaffected portion of tumor nephrectomy). We grouped the tissue samples based on the histological readings of the kidney biopsies. Samples with evidence of glomerular and tubulointerstitial fibrosis were assigned into the diseased group (Table 1). The research population was notable for its diversity in both ethnicity and etiology of renal disease, but their age, body weight, BMI and renal function showed no statistical differences. Majority of the participants had mild, Stage 3 CKD. The kidney tissue was microdissected into glomerular and tubulointerstitial fractions and AffymetrixU133 expression arrays were performed separately [24]. Quality of the microdissection was determined by evaluating the expression of glomerular specific genes (NPHS1, NPHS2, and SYNPO). We included 52 good quality hybridizations into our analysis.

**Table 1:** Demographics of the research participants.

Characteristics	Healthy females	Healthy males	p	Diseased females	Diseased males	p
	(n = 10)	(n = 9)		(n = 14)	(n = 9)	
**Age (years)**	54.3±13.2	55.3±17.5	0.9	51.0±18.5	49.0±31.3	0.85
**Ethnicity**						
**Asian Pacific Islander**	0	0		2	0	
**White, non-Hispanic**	1	2		1	0	
**Black, non-Hispanic**	5	5		4	3	
**Hispanic**	0	1		2	1	
**Other & Unknown**	4	1		5	5	
**Weight, (kg)**	77.8±18.0	76.3±16.8	0.86	84.1±26.8	72.0±26.0	0.35
**BMI (kg/m2)**	26.9±5.0	23.2±4.0	0.14	32.4±10.6	26.0±7.3	0.16
**HTN**	1 (10%)	5 (55.6%)	0.06	8 (57.1%)	4 (44.4%)	0.68
**DM**	0	0		8 (57.1%)	5 (55.6%)	
**Proteinuria (dipstick)**						
**Negative**	7	7		5	1	
**30 mg/dL**	2	1		1	4	
**100 mg/dL**	0	0		4	3	
**Unknown**	1	1		4	1	
**Serum BUN (mg/dL)**	13.7±3.5	15.1±4.0	0.43	21.5±15.8	22.0±17.7	0.91
**Serum creatinine (mg/dL)**	1.0±0.3	1.2±0.3	0.06	2.2±2.3	1.0±1.1	0.38
**eGFR (ml/min)**	78.0±32.7	77.9±16.5	0.99	57.6±47.0	78.0±56.8	0.4
**Histology**						
**Normal**	10	9		0	0	
**Diabetic Nephropathy**				5	0	
**FSGS**				2	6	
**Arteriosclerosis**				1	0	
**Global glomerulosclerosis**				4	0	
**Fibrosis**				1	2	
**Unknown**				1	1	

Abbreviations used: BMI: Body Mass Index; DM: Diabetes Mellitus; BUN: Blood Urea Nitrogen; FSGS, Focal Segmental Glomerulosclerosis. eGFR (estimated glomerular filtration rate) was calculated using the MDRD formula. Student's t-test was used to determine the statistical significance between sexes for age, weight, BMI, BUN, creatinine, eGFR.

**Figure 2:**
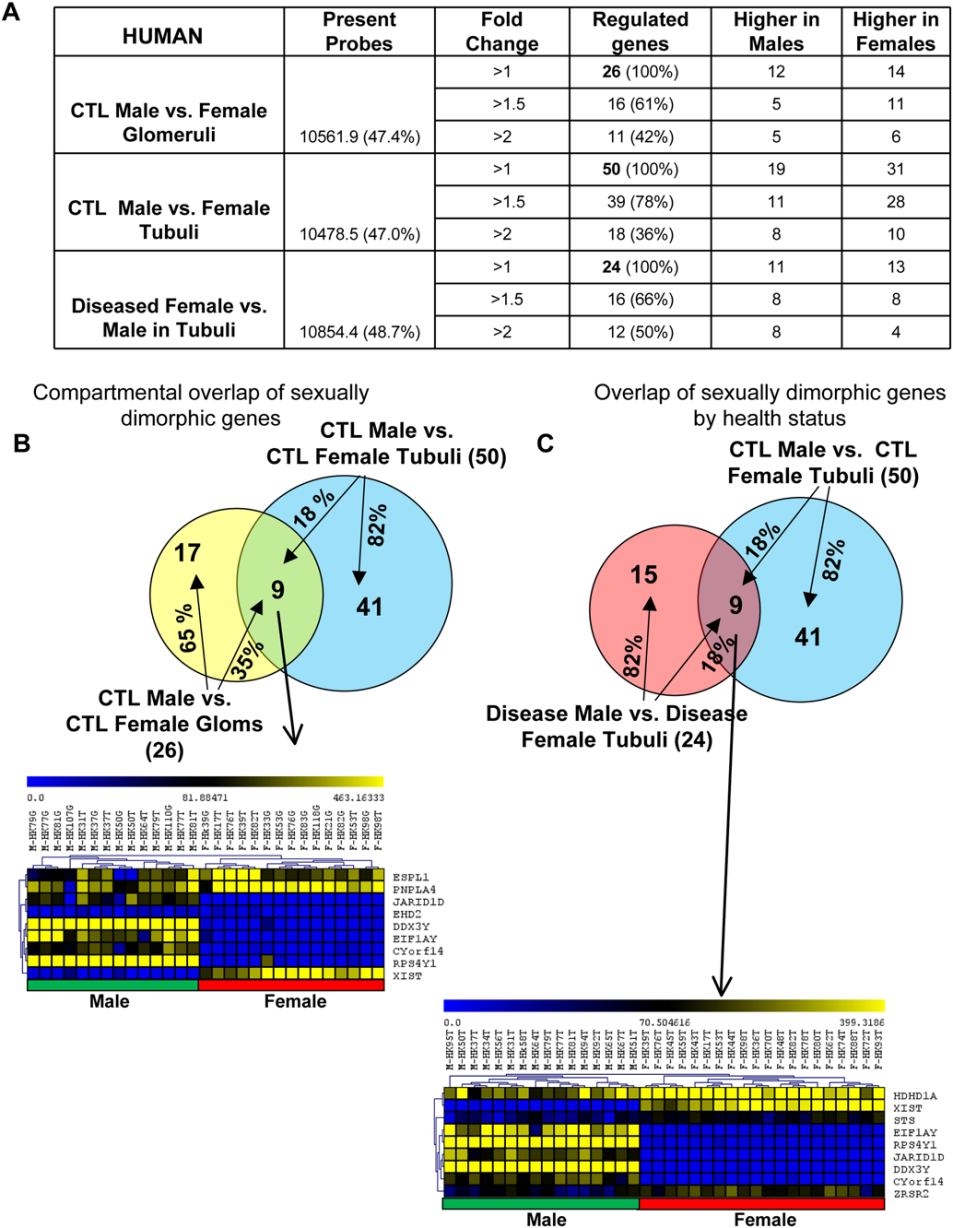
Gene expression changes in control and diseased human kidney samples. (A) Distribution of genes that are differentially expressed in male and female human kidneys in glomerular and tubulointerstitial compartments (p<0.01, Student's t-test). The value in the parenthesis following the number of dimorphic gene shows the percentage of active probes. (B) Upper panel: the overlap of sexually dimorphic genes when tubuli were compared to glomeruli from healthy kidneys; Lower panel: hierarchical clustering (complete linkage) of the overlapping genes identified between healthy glomeruli and tubuli. (C) Upper panel: the overlap of sexually dimorphic genes when diseased tubuli were compared to healthy tubuli. Lower panel: hierarchical clustering (complete linkage) of the overlapping genes between diseased and healthy tubuli. In the Venn's diagram, the gene number in individual distinct area and the percentage over the total gene number in individual group are shown. In the gene clusters, one row represents one gene and one column represents one sample. The yellow color indicates higher gene expression level, while the blue one indicates lower level.

First, we determined gene expression differences between healthy male and female microdissected glomeruli. Statistical analysis (Student t-test, p<0.01) identified 26 differentially regulated transcripts in healthy glomeruli (Figure 2A, Table S4A). Among them, 16 genes had sex-specific expression levels at a fold-change threshold >1.5 and 11 genes showed >2-fold differential expression level between sexes. Statistical analysis identified 50 differentially expressed transcripts between sexes in the tubulointerstitial compartment (Figure 2A and Table S4B). Out of the 50 sex-specific genes, 39 genes showed >1.5-fold differential expression and 18 genes showed >2-fold differential expression between sexes (Figure 2A). The small number of sex biased genes with higher fold changes might be due to the greater sample heterogeneity. We found 9 (24–37% of total) sex-biased transcripts differentially regulated in both glomeruli and tubuli (Figure 2B) i.e. regardless of the kidney compartment. These transcripts were: EHD2, DDX3Y, EIF1AY, CYorf14, ESPL1, JARID1D, PNPLA4, RPS4Y1, and XIST.

In addition, we also used the SAM analysis (with a very stringent FDR of 0%) to define gene expression differences between healthy male and female kidneys. Firstly, 5 transcripts were identified to be differentially expressed in glomeruli between male and females and they all showed more than 2 fold change (Table S9 A). Secondly, 8 transcripts were found to be differentially expressed in tubuli between male and females (Table S9 B). We found 4 transcripts that were commonly differentially expressed in both glomeruli and tubuli, these transcripts were XIST, JARID1D, RPS4Y1 and DDX3Y.

In order to investigate whether there is a sex-specific renal injury response in the human kidney regardless of the type of the renal disease, we compared gene expression differences between diseased male and female tubuli, reasoning, that while the glomerular morphology shows significant differences in various glomerular diseases, to our current knowledge one cannot distinguish tubulointerstitial fibrosis based on the primary disease. We found 24 transcripts differentially expressed in the tubulointerstitial compartments (Student t-test, p<0.01) when male samples were compared to female samples under disease condition (Figure 2A, Table S5). Nine genes; XIST, DDX3Y, JARID1D, CYorf14, EIF1AY, HDHD1A, RPS4Y1, STS, and ZRSR2 showed differential expression both in healthy and diseased human kidneys (Figure 2C). The lack of significant overlap between the baseline and disease related gene expression might indicate that there are not only baseline differences but a sexually dimorphic response during disease.

When the SAM analysis (with a very stringent FDR of 0%) was used, we found 8 differentially expressed transcripts in tubulointerstitial fraction between male and female under disease condition (Table S10). We identified 6 common sex-biased transcripts when comparing sex-biased transcripts in tubulointerstitial compartment under baseline and disease condition, these are XIST, ZRSR2, CYorf14, JARID1D, DDX3Y and RPS4Y1.

An important limitation of our studies is the use of human biopsies from a heterogenous population with heterogenous kidney disease. A significant confounder might also be related to different sex hormone levels in pre and post-menopausal women. To address this issue we compared gene expression changes in women less than 49 years of age (42±3, n = 4) to those 50 and above (67±9, n = 4). We found 36 differentially expressed transcripts in the glomeruli and 361 transcripts that were differentially expressed in the tubulointerstitium (Table S6). This analysis showed significant difference in estrogen receptor 1 and 2 expressions when samples from participants under 49 were compared to those over 50, indicating the role of sex hormones. However, (given the cross sectional design) we can not assess whether the regulation of these genes correlates with hormonal status (pre vs. post-menopausal status) or by aging.

To further dissect the role of sex hormones we also made comparisons between healthy male and female glomerular and tubular samples obtained from participants <49 years of age. We found 35 differentially expressed genes when healthy male glomeruli were compared to female glomeruli. Interestingly 4 of the 35 genes (GNAS, ID2, TJAP1, COL4A6) had androgen receptor binding sites on their promoter. Similar analysis performed on tubular samples of participants <49 years of age identified 93 differentially expressed genes (however there were only 2 samples in each subgroup).

### The species-specificity of sexually dimorphic genes

**Figure 3:**
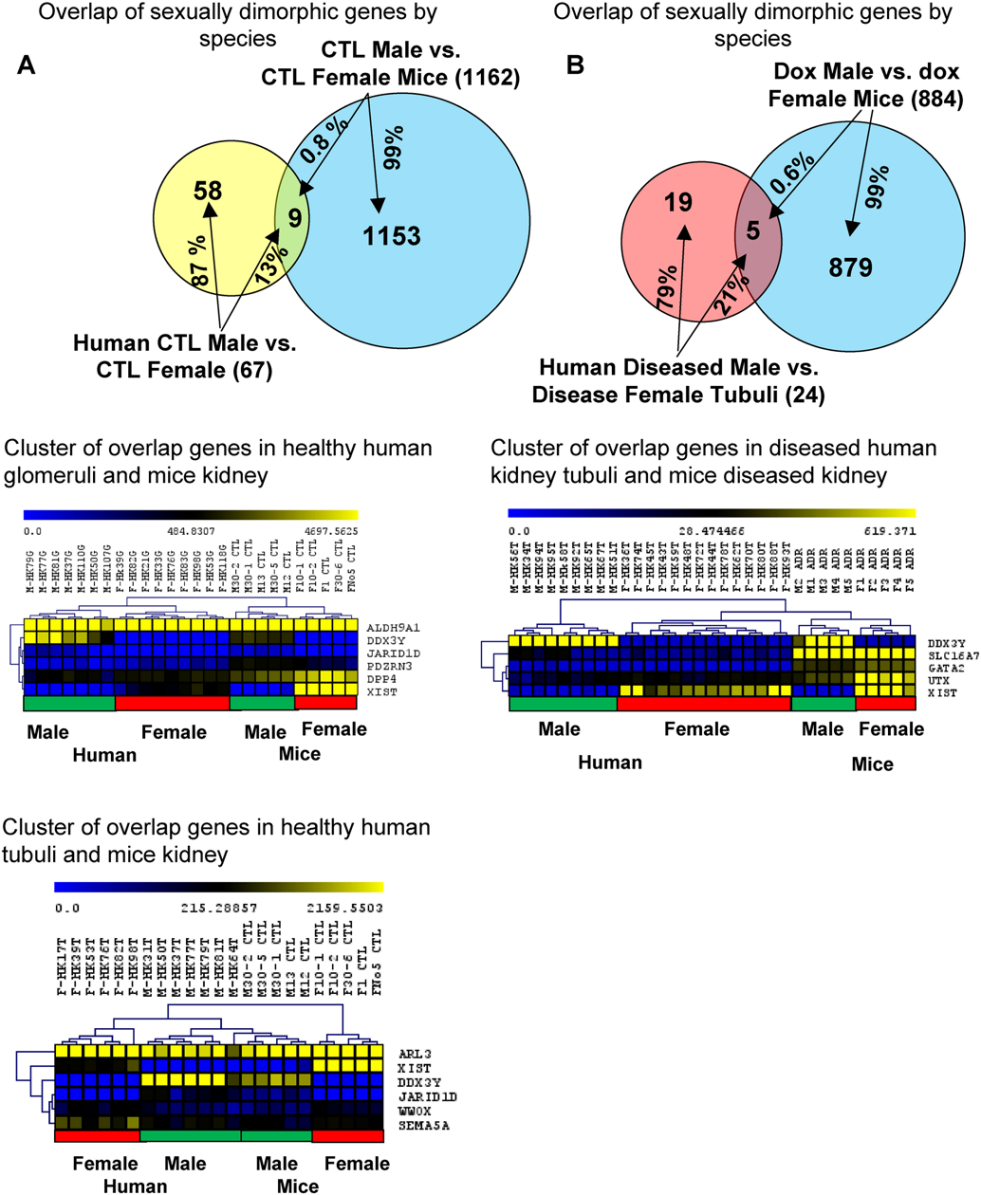
Species specific gene expression differences. (A) Upper panel: overlap of sexually dimorphic genes in kidneys of healthy people and mice; Lower panel: hierarchical clustering (complete linkage) of the overlapping genes identified between healthy human glomeruli/tubuli and control mice. (B) Upper panel: overlap of sexually dimorphic genes of diseased human kidneys and diseased murine kidneys. Lower panel: hierarchical clustering (complete linkage) of the overlapping genes identified between diseased human and murine kidneys.

Next we examined the concordance of human and murine gender biased genes in the kidney. The tubulointerstitium constitutes the majority (>95%) of the kidney, therefore we compared gene expression levels in whole kidney lysates of mice to transcript levels of the tubulointerstitial fraction of human kidneys. When we compared 67 sex-biased genes in “healthy” human kidneys with 1162 sexually dimorphic genes in the murine kidney (Figure 3A), we identified 9 transcripts that were differentially regulated in both species (corresponding to 13% of all human gender biased genes and 0.8% of all murine gender biased genes). These genes were: ALDH9A1, ARL3, DDX3Y, DPP4, JARID1D, PDZRN3, SEMA5A, WWOX and XIST.

We also compared the sexually dimorphic genes in murine and human diseased kidneys. This analysis again identified a handful of commonly regulated genes; DDX3Y, GATA2, SLC16A7, UTX and XIST transcripts were differentially regulated in both human and murine diseased kidneys (Figure 3B). Therefore our results indicate significant species-specific differences in gender biased gene expression levels in the kidney.

### Functional categories of sexually dimorphic genes

In order to understand the function of the sexually dimorphic genes in the kidney we used the David 2.0 web-based software (http://david.abcc.ncifcrf.gov) to find significantly enriched functional groups. To avoid the functional redundancy we used the level5 gene ontology (GO) annotation. For the biological process group we identified 5 functional groups (2 in glomeruli and 3 in tubuli) with statistically significant enrichment in healthy human kidney (Table 2 and Table S7). These groups included translation, translation initiation, protein-RNA complex assembly and angiogenesis. Sexually dimorphic genes in the human kidney were more likely to encode cytosolic proteins (Table 2 and Table S7).

**Table 2:** Gene ontology groups of gender biased genes in human kidneys.

Gene ontology	Sex-biased genes in healthy human glomeruli	sex-biased genes in healthy human tubuli	sex-biased genes in human diseased Tubuli
Biological process	regulation of angiogenesis	protein-RNA complex assembly	none
	translation	regulation of progression through cell cycle	
		translational initiation	
Molecular function	none	none	none
Cellular component	cytosolic small ribosomal subunit	none	none
	cytosol		
	cytoplasmic part		
	cytoplasm		

Statistically significantly overrepresented gene ontological terms (at level 5 terms) were identified in biological process and cellular component, whereas no overrepresented gene ontological term at level 5 was found in molecular function. David 2.0 program was used to identify the overrepresented gene functional groups and the statistical significance was determined by modified Fisher's exact test (p<0.05).

Functional annotation analysis of murine sexually dimorphic genes identified 58 and 39 GO terms in the biological process group with statistically significant enrichment in control and diseased kidneys, respectively. The top GO terms in the healthy kidney included steroid and fatty acid synthesis, lipid (glycolipid, and sphingolipid) metabolism, actin polymerization and vascular developmental pathways (Table 3 and Table S7). Dimorphic genes in diseased animals belonged not only to the lipid (phospholipid) biosynthesis, but to intracellular (vesicle mediated) transport process as well (Table S7).

**Table 3:** Functional categories of sexually dimorphic genes in murine kidneys.

Gene ontology	Sex-biased genes in mice CTL kidney	sex-biased genes in mice diseased kidney
Biological process	monocarboxylic acid metabolic process	lipid biosynthetic process
	fatty acid metabolic process	monocarboxylic acid metabolic process
	lipid biosynthetic process	fatty acid metabolic process
	glutathione metabolic process	mRNA metabolic process
	proteolysis	carboxylic acid biosynthetic process
	acetyl-CoA metabolic process	serine family amino acid metabolic process
	carboxylic acid biosynthetic process	fatty acid biosynthetic process
	modification-dependent macromolecule catabolic process	positive regulation of transcription
	blood coagulation	positive regulation of nucleic acid metabolic process
	protein catabolic process	positive regulation of transcription, DNA-dependent
Molecular function	unspecific monooxygenase activity	glucuronosyltransferase activity
	glucuronosyltransferase activity	symporter activity
	iron ion binding	iron ion binding
	acyltransferase activity	pyrophosphatase activity
	symporter activity	glyceraldehyde-3-phosphate dehydrogenase activity
	metalloexopeptidase activity	threonine endopeptidase activity
	aminopeptidase activity	unspecific monooxygenase activity
	sodium ion binding	sugar transmembrane transporter activity
	guanylate kinase activity	glutathione peroxidase activity
	sugar transmembrane transporter activity	
Cellular component	cytoplasm	cytoplasm
	cytoplasmic part	cytoplasmic part
	mitochondrion	intracellular membrane-bound organelle
	vesicular fraction	intracellular organelle
	microsome	mitochondrion
	cytosol	vesicular fraction
	endoplasmic reticulum	mitochondrial part
	intracellular organelle	microbody
	cytosolic ribosome (sensu Eukaryota)	peroxisome
	mitochondrial part	mitochondrial inner membrane

Overrepresented gene ontological terms (at level 5 terms) were identified in biological process, molecular function and cellular component. David 2.0 program was used to identify the overrepresented gene functional groups and the statistical significance was determined by modified Fisher's exact test (p<0.05). Only top 10 GO terms based on the p value ranking were shown in the table.

### Species-specific chromosomal enrichment

Mammalian sex chromosomes are enriched for sexually dimorphic genes, which are involved in sex development and differentiation [25], [26]. Thus, in the present study we analyzed the chromosomal localization of renal sexually dimorphic genes by using GeneTrail Software (http://genetrail.bioinf.uni-sb.de [27]). This Software compares the proportion of genes in a geneset located on a specific chromosome to the general chromosomal distribution of all the genes. The list of genes differentially regulated in control and diseased human kidneys only showed significant enrichment for X and Y chromosomes (Table 4), while the differentially expressed genes in the mouse kidney showed enrichment not only for sex chromosomes but for various autosomes (including chromosome 4, 7, 14, 19) (Table 4). Interestingly previous studies already indicated male biased gene enrichment in the liver on chromosome 19 [21]. Thus, our results indicate a conserved enrichment for sex chromosomes and species-specific chromosomal enrichment to autosomes.

**Table 4:** Chromosomal distribution of gender biased genes.

	Human (glom+tubule)	Mouse (whole kidney)
Healthy male vs. female	X, Y	X,Y,3,4,6,7,8,14,19
Diseased male vs. female	X, Y	X,4,5,7,9,14,16,19

Enriched chromosomes for the sexually dimorphic genes in kidneys of human and mouse. The GeneTrail Software was used in this study (p<0.05, Fisher's exact test).

### Enrichment of transcription factor binding sites (TFBS) in response to disease

In order to understand the regulation of sexually dimorphic genes in human and mouse kidneys, we examined transcription factor binding sites (TFBS) on the sex biased transcripts. With the use of the oPOSSUM program [28], which contains around 100 verified transcription binding sites in its database, we searched for regulatory elements within 5 kb upstream regions of the sexually dimorphic genes, which could be indicative of transcription factor binding.

In healthy human kidney tubules, sex-biased genes showed significant enrichment for several TF, including: ELK4, Broad-complex_1 & 3, CF2-II, RORA1, NR2F1 and CF2-II transcription factor-binding sites (Table 5). The promoter region of the murine sexually dimorphic genes showed enrichment for 7 different TF under baseline condition (Table 6 and Table S8). Based on the known effects of sex steroids, we were specifically interested in finding binding sites for estrogen or androgen receptors and transcription factors regulated by sex hormones. Analysis with the oPOSSUM program indicated no statistically significant enrichment for sex steroid hormone receptor binding sites on the promoter of the sexually dimorphic genes.

**Table 5:** TFBS of gender biased genes in human kidneys.

TFBS in control male vs control female human kidney	TFBS in diseased male vs. diseased female Human kidney
ELK4 (ETS)	Broad-complex_1 (Zn-Finger)
Broad-complex_3 (ZN-Finger)	RORA1 (Nuclear receptor)
CF2-II (Zn Finger)	NR2F1 (Nuclear receptor)
	CF2-II (Zn-Finger)

Overrepresented TFBS identified in the sex-biased genes from human kidneys. The statistical significance was determined by Fisher's exact test (p<0.05). The oPPOSUM Sotware was used to identify TFBS.

**Table 6:** TFBS of gender biased genes in murine kidneys.

TFBS in control male vs. female mouse kidney	TFBS in diseased male vs. disease female mouse kidney	
HNF1A	HNF1A	Broad-complex_3
Broad-complex_3	FOXF2	Foxa2
Lhx3	Broad-complex_4	TBP
SRY	NFIL3	PEND
hb	SRY	Broad-complex_1
Foxd3	Lhx3	NR1H2-RXRA
Broad-complex_4	MYB.ph3	FOXI1
	GAMYB	GABPA
	PBX1	HMG-IY
	PBF	IRF1
	MNB1A	hb
	Foxq1	bZIP911
	Foxd3	SQUA
	Dof2	Cebpa
		ELK4

Overrepresented TFBS identified in the sex-biased genes from mice kidneys. The statistical significance was determined by Fisher's exact test (p<0.05). The oPPOSUM Sotware was used to identify TFBS.

We also compared TFBS of sex-biased genes: in healthy kidneys there was a common enrichment for Broad-complex_3 binding sites, while in diseased kidneys, there was enrichment for transcription factors Broad-complex_1. Thus our results suggest that human and mouse male and female kidneys exhibit some degree of species-independent enrichment of TFBS.

## Discussion

Understanding sex related differences in renal disease development is a critical but understudied issue. The incidence of ESRD is 50% higher in males, which makes gender one of the most significant risk determinants for the development of kidney disease. The cause and mechanism of sex bias in renal disease development is largely unknown. This study was aimed to determine global gene expression differences in male and female kidneys of humans and mice in order to better understand gender differences.

According to our knowledge this is the first study to describe gender related global gene expression differences in human kidney samples. In the present study we used 42 human kidney samples and identified 67 (26 in glomeruli and 50 in tubules) transcripts differentially expressed in “healthy” human kidneys. We not only analyzed control kidneys but also found 24 differentially expressed transcripts when diseased male and female tubulointerstitial samples were compared. We think that the relatively small number of differentially expressed genes with higher fold changes may be related to the high degree of sample heterogeneity (including race and type of renal disease).

Our study shows that the most consistently identified gender biased genes (found both in human and mice, control and diseased kidneys) are those that localized on sex chromosomes (X, Y). These genes largely, but not fully overlap with genes that have been described in other organs. Tissue specific expression of sex chromosome genes has been observed in somatic tissues, but their role is largely unknown [25], [26], [29], [30]. During development, Y-chromosome related genes are involved in male specification. The differential expression of X, Y-chromosome genes might also be required to restore balanced expression of X-linked genes between the sexes. However, this general mechanism cannot explain the tissue specific expression of some of the sex chromosome genes. Therefore we might speculate that this tissue specific expression could be important for gender differences that can be observed in certain disease conditions but not in others.

The role of many of the genes located on the sex chromosomes is unknown. However, we would like to highlight a recent paper by Tan et al. [31]. In this study, the authors determined that antibodies against RPS4Y1 and DDX3Y were associated with increased rate of acute rejection when a male kidney was transplanted into a female recipient. RPS4Y1 and DDX3Y were some of the top genes that we identified as differentially expressed when we compared male and female kidneys, indicating that sex chromosomal genes might have important clinical significance. Further mechanistic studies are needed to examine this concept.

A surprising and important finding of our studies is the narrow concordance between gender biased genes in mice and humans (both at baseline and under disease conditions). This degree of overlap might not be statistically different from random variations. Given that many of the gender biased genes were sex chromosome related and there is an almost 10-fold difference in the number of known transcripts in the human and murine sex chromosomes, this might not be unexpected. Our results strongly reinforce the concept that caution needs to be exercised when we use rodent models to study gender related differences.

Interestingly, there was no clear enrichment for sex steroid hormone receptor binding sites on the promoter of the gender-biased genes either in human or in murine kidneys. While one can argue that some of the human samples were from post-menopausal women, it is important to note that the same observation was true for the murine samples as well. The absence of clear enrichment for sex-hormone regulated genes is consistent with the results 2 previous gene expression studies performed on mice [19], [21]. This could indicate that genes are regulated in a more complex regulatory network, rather than direct sex steroid hormone binding. Genomic and nongenomic actions of sex steroid hormones might converge on the regulation of target genes via signal transduction pathways that modulate the activity of several transcription factors. In addition, ovarian steroids are released in a cyclic fashion, thus their target genes might also be expressed in a cyclic fashion, making it increasingly difficult to identify them. Gene expression studies aiming to reveal cycle-dependent changes are in their infancy. Sex hormones are the main focus of research to decipher the mechanism of sex differences, but increasing amount of evidence suggest that gender differences can also be mediated by growth hormone, which has a different secretion pattern in men and women [32], [33], [34].

Our study is one of the first that analyzed gender related global gene expression changes in renal injury models. We determined that while there were baseline gender specific gene expression differences in the kidney, different genes were differentially expressed in diseased kidneys. This observation might have very important consequences for clinical medicine and could imply that different treatment strategies need to be applied for men and women with renal disease. These results are consistent with findings obtained in the cardiovascular field, where gender specific differences have been reported for disease development and treatment response. Unfortunately, there were a few inherent limitations of our study. First, we could not distinguish whether the severity of renal disease resulted from systemic differences (including drug metabolism) or from gene expression response differences inherent to the kidney. In addition, the cross-sectional design of our study did not allow us establish causality. Our analysis also included multiple comparisons, which increases the probability of false discoveries. We were cognizant of this issue; however, as this was one of the first studies that explored sexually dimorphic gene expression changes in human and murine kidneys we opted to maximize the sensitivity at the expense of specificity. Further large scale expression and mechanistic studies will be necessary to confirm our finding and to establish causality.

In summary, here we provide a first description of sex specific gene expression differences in human and murine kidneys under baseline and disease conditions. Our studies highlight significant differences in human and murine kidneys both at baseline and in their response to injury. We identified several new candidate transcripts that could enable us to better understand sex specific differences in the occurrence and pharmacologic responses of kidney injury in humans. Our studies suggest a need to further investigate sex specific treatments for kidney disease.

## Materials and Methods

**The clinical study** used the cross-sectional design. Kidney samples were obtained at the Montefiore Medical Center (MMC) of the Albert Einstein College of Medicine (AECOM) from living allograft donor and surgical nephrectomies and from left-over portions of diagnostic kidney biopsies. The study was approved by the Institutional Review Board of the AECOM and MMC (2002-202 to K.S.). Written consent was obtained from all living kidney donors and recipients. Clinical information was collected using standardized datasheets.

### Tissue handling

Kidney tissue was obtained in the operating room immediately after removal from the patient. Tissue was placed into RNALater right after removal from the body and was kept at 4°C. Part of the sample was fixed in formalin and embedded in paraffin. 4 µm sections were cut and stained with PAS and were evaluated and graded by an expert nephropathologist.

### Microdissection

1 mm×1 mm biopsy tissues were placed under Olympus model SZX12 stereomicroscope using 90×magnification. The biopsy tissue was manually microdissected at 4°C in RNALater for glomerular and tubular compartment using fine tip forceps. In general, 5 glomeruli that readily released from the capsule were collected and placed into cold RLT solution (Qiagen RNeasy kit). The corresponding tubulointerstitial and vascular compartment was placed into RLT solution also. For easier designation we called this component tubules throughout the manuscript. Dissected tissue was homogenized using Powergen125 (Fisher) homogenizer and stored at −80°C. RNA was prepared using RNAeasy mini columns (Qiagen, Valencia, CA) according to manufacturer's instruction. RNA quality and quantity was determined using Lab-on-Chip Total RNA PicoKit, Agilent BioAnalyzer. Only samples without evidence of degradation were further used.

**Animal Experiments** were conducted in accordance with the Guide for the Care and Use of Laboratory Animals and were approved by the Institutional Animal Care and Use Committee of the Albert Einstein College of Medicine. We made all efforts to minimize the number of animals used and their suffering.Renal disease was induced in about 10-week old male and female Balb/c mice via intravenous injection of 12 mg/kg doxorubicin. Urine was collected in metabolic cages for 24 hrs and albuminuria was measured using mouse albumin specific ELISA (Bethyl Laboratories), and corrected to urinary creatinine excretion, by using picric acid method.

**Microarray Procedure** was compliant with MIAME. For the human kidney tissue (glomeruli or tubuli), purified total RNAs were amplified using the Two-Cycle Target Labeling Kit (Affymetrix) as per manufacturer's protocol. Briefly, total RNA (10 ng) from each sample was first reverse transcribed into cDNA using a T7 promoter-dT primer [5′GGCCAGTGAATTGTAATACGACTCACTATAGGGAGGCGG-(T)24], then converted to double-stranded cDNA and amplified with an in vitro transcription reaction using T7 RNA polymerase. The products were then reverse transcribed into cDNA again, using random hexamer primers. A final in vitro transcription reaction was performed using the GeneChip IVT labeling kit (Affymetrix, USA) to produce biotinylated cRNA for microarray.

Mouse tissue total RNA was prepared from whole kidneys using Trizol® (Invitrogen). Gene expression studies were performed using the Affymetrix One Cycle labeling kit as per manufacturer's instruction.

For the analysis of gene expression data after hybridization and scanning, raw data files were imported into Array Assist Software (Invitrogen, USA). Database and expression levels were normalized using the GCRMA algorithm. This normalization method is a mathematical technique used to reduce discrepancies in hybridization patterns that might result from variables in target amplification, hybridization conditions, staining or probe array lot. Normalizations standardize the data to facilitate identification of genuine gene expression difference. For statistical analysis, the data was exported into Excel (Microsoft) and Statistical Analysis of Microarray Software and Student's t-test were used. Gene expression data is uploaded to NICBI gene expression omnibus (GEO numbers; GSE12682, GSE12683).

**Histology** was evaluated in formalin fixed paraffin embedded kidney tissues, which were stained using the PAS protocol.

### Gene Ontology Classification and Overrepresentation of Biological Themes

All significant gene entries were subjected to GO classification. Significant overrepresentation of GO-classified biological processes was determined by comparing the number of genes in the biological process that were significantly differentially expressed in a particular mouse strain to the total number of genes relevant to that biological process printed on the array using the publicly available DAVID 2.0 software (http://david.abcc.ncifcrf.gov/). The significance was determined by modified Fisher's exact test (EASE Score, p<0.05).

**Analysis of overrepresented chromosomes** was performed using GeneTrail Software [27] (http://genetrail.bioinf.uni-sb.de/). The significance was determined by Fisher's exact test (p<0.05).

**Analysis of enrichment for TFBS** was performed by using the oPOSSUM Sotware [28] (http://burgundy.cmmt.ubc.ca/oPOSSUM/). For each transcript, the top 10% of conserved regions in the 5000 bp up or down-stream sequences with minimum conservation of 70% and matrix match threshold of 80% were scanned for TFBS and the significance level was determined by Fisher's exact test score at p<0.05.

## Supporting Information

Table S1 List of differentially expressed transcripts in kidneys of healthy Balb/c male (M) and female (F) mice (n = 5/per group). Statistical significance was determined by using the SAM analysis (FDR<0.3%) Ratio of gcRMA normalized relative mRNA expression levels (Ratio F/M and Ratio M/F)
(1.06 MB XLS)

Table S2 List of differentially expressed transcripts in kidneys of doxorubicin treated Balb/c male and female mice (n = 5/per group). Statistical significance was determined by using SAM analysis (FDR<0.3%) Ratio of gcRMA normalized relative mRNA expression levels (Ratio F/M and Ratio M/F)
(0.81 MB XLS)

Table S3 sheet A. List of differentially expressed transcripts in kidneys of control and doxorubicin treated male Balb/c mice (n = 5/per group) Statistical significance was determined by using SAM analysis (FDR<3%) Ratio of gcRMA normalized relative mRNA expression levels (Ratio of doxorubicin vs control kidneys Dox/CTL) sheet B. List of differentially expressed transcripts in kidneys of control and doxorubicin treated female Balb/c mice (n = 5/per group) Statistical significance was determined by using SAM (FDR<4%) Ratio of gcRMA normalized relative mRNA expression levels (Ratio of doxorubicin vs control kidneys Dox/CTL)
(0.07 MB XLS)

Table S4 A Differentially expressed genes between sexes in human healthy glomeruli. Gene expression values in healthy glomeruli of females (F) and males (M) were averaged to calculate the ratio (F/M), and the statistical significance was determined using Student's t-test. Only the genes with p value<0.01 were considered significant. B. Differentially expressed genes between sexes in human healthy kidney tubulointerstitium. Gene expression values in healthy tubuli of females and males were averaged to calculate the ratio (F/M), statistical significance was determined using Student's t-test. Only the genes with p value<0.01 were considered significant.
(0.03 MB XLS)

Table S5 Differentially expressed genes between sexes in human diseased kidney tubulointerstitium. Gene expression values in diseased tubuli of females and males were averaged to calculate the ratio (F/M), statistical significance was determined by Student's t-test. Only the genes with p value<0.01 were considered significant.
(0.02 MB XLS)

Table S6 A List of differentially expressed transcripts of healthy human female glomeruli of <49 vs. >50 years of age (n = 4/per group). Statistical significance was determined by Student's t-test (genes with p value<0.01) Ratio of gcRMA normalized relative mRNA expression levels (<50/>50) B. List of differentially expressed transcripts of healthy human female tubuli of <49 vs. >50 years of age (n = 4/per group). Statistical significance was determined by Student's t-test (genes with p value<0.01) Ratio of gcRMA normalized relative mRNA expression levels (<50/>50)
(0.09 MB XLS)

Table S7 The complete list of gene ontology terms showing significant enrichment. Enriched GO terms at level 5 were determined by David 2.0 program (p<0.05, modified Fisher Exact test). Gene Ontology terms were determined for Biological process/Molecular Function and Cellular Component. A. Gender biased genes healthy human male glomeruli vs healthy female glomeruli B. Gender biased genes healthy human male tubuli vs healthy female tubuli C. Gender biased genes diseased human male tubuli vs diseased female tubuli D. Gender biased genes healthy male Balb/c mice vs healthy female mice E. Gender biased genes diseased doxorubicin treated male Balb/c mice vs dox treated female mice
(0.03 MB XLS)

Table S8 The complete list of transcription factor binding sites (TFBS) showing statistically significant enrichment on the gender-biased genes. A. Human healthy male vs female tubuli B. Human diseased male vs diseased female tubuli C. Healthy male vs female Balb/c mice D. Dox treated male vs dox treated female Balb/c mice. The oPPOSUM Software was used to generate this list.
(0.02 MB XLS)

Table S9 Sheet A. The complete list of differentially expressed genes between sexes in human healthy glomeruli. Gene expression values in healthy glomeruli of females (F) and males (M) were averaged to calculate the ratio (F/M). Statistical significance was determined by using the SAM test (FDR = 0%). Sheet B. Differentially expressed genes between sexes in human healthy kidney tubulointerstitium. Gene expression values in healthy tubuli of females and males were averaged to calculate the ratio (F/M). Statistical significance was determined by using the SAM test (FDR = 0%).
(0.02 MB XLS)

Table S10 The list of differentially expressed genes between sexes in human diseased kidney tubulointerstitium. Gene expression values in diseased tubuli of females and males were averaged to calculate the ratio (F/M). Statistical significance was determined by using the SAM test (FDR = 0%).
(0.02 MB XLS)
